# Lipids around the Clock: Focus on Circadian Rhythms and Lipid Metabolism

**DOI:** 10.3390/biology4010104

**Published:** 2015-02-05

**Authors:** Davide Gnocchi, Matteo Pedrelli, Eva Hurt-Camejo, Paolo Parini

**Affiliations:** 1 Division of Clinical Chemistry, Department of Laboratory Medicine, Karolinska Institutet at Karolinska University Hospital Huddinge, Stockholm, 14186, Sweden; E-Mails: davide.gnocchi@ki.se (D.G.); matteo.pedrelli@ki.se (M.P.); 2 Strategy and Externalization, CVMD iMED, AstraZeneca, R&D, Mölndal, SE-431 83, Sweden; E-Mails: Matteo.Pedrelli@astrazeneca.com (M.P.); Eva.Hurt-Camejo@astrazeneca.com (E.H.C.)

**Keywords:** circadian clock, central oscillator, peripheral oscillators, lipid metabolism, lipid transport, metabolic diseases

## Abstract

Disorders of lipid and lipoprotein metabolism and transport are responsible for the development of a large spectrum of pathologies, ranging from cardiovascular diseases, to metabolic syndrome, even to tumour development. Recently, a deeper knowledge of the molecular mechanisms that control our biological clock and circadian rhythms has been achieved. From these studies it has clearly emerged how the molecular clock tightly regulates every aspect of our lives, including our metabolism. This review analyses the organisation and functioning of the circadian clock and its relevance in the regulation of physiological processes. We also describe metabolism and transport of lipids and lipoproteins as an essential aspect for our health, and we will focus on how the circadian clock and lipid metabolism are greatly interconnected. Finally, we discuss how a deeper knowledge of this relationship might be useful to improve the recent spread of metabolic diseases.

## 1. Introduction

A properly functional metabolism is one of the key factors determining our wellbeing. Metabolism and transport of lipids and lipoproteins have been deeply investigated by scientists in the past decades, and it is now widely accepted that defects in their regulation are strongly associated with the development of many pathological conditions, such as the metabolic syndrome, most cardiovascular diseases, and even tumour development [1].

The first pioneers who commenced the study of biological rhythms were not initially well considered by the scientific community. The topic was perceived too “exotic” to be worthy of consideration, and the idea of the existence of a molecular clock that oscillates autonomously, being synchronised by external stimuli such as light, was difficult to accept. Despite this initial diffidence, this subject is now drawing the interest of researchers, especially for its relevance in regulation of metabolism [2].

Here we first give an overview on the discovery of the circadian clock, and on its organisation from an anatomical and biochemical point of view. Then we will focus on the deep relationship between the circadian clock and energy metabolism, pointing to lipid and lipoprotein metabolism and transport as key determinants of our health.

## 2. The Circadian Clock

### 2.1. Historical Overview

The existence in higher eukaryotes of an internal periodicity, independent from external stimuli such as light, was long ago recognised by the French scientist Jean-Jacques d’Ortous De Mairan, in the beginning of the 18th century. He discovered that the plant *Mimosa Pudica*, even when closed in a wood locker without any source of light, retained the usual 24 h periodicity in the opening-closing cycles of its leaves [3]. About one hundred years after that discovery, in the middle of 19th century, the Swiss botanic Alphonse de Candolle, using the same plant, assessed that the leaf cycle was not constant, being slightly different from 24 h [4]. But it was not until the early middle of the 20th century—but published later—that a rigorous confirmation would have come: the German scientist Erwin Bünning discovered that the leaf cycle of *Phaseolus*—the common bean—showed an average periodicity of 24.4 h [5]. Later, the British born biologist Colin Pittendrigh and his colleagues found that similarly, in *Drosophila* the daily cycle was not exactly 24 h, but it oscillated between 22 h and 28 h. Those findings introduced a key concept for the understanding of biological rhythms: the periodicity is not induced by external stimuli, but it is “innate”, with an average period slightly different from 24 h. This internal “pendulum” that oscillates autonomously was termed “free-running” rhythm, and the external environmental stimuli are just able to synchronise it. Light is the principal “timer” (“Zeitgeber” in German) that regulates the free-running cycle, and the effect of synchronisation is known as “entrainment”. Light synchronises mammalian circadian rhythms with the environmental time by modulating retinal input to the central nervous system (CNS), and this photic entrainment requires neither rods nor cones. In fact, retinal ganglion cells innervating the CNS are intrinsically photosensitive, and depolarise in response to light even when all synaptic inputs from rods and cones are blocked [6]. These ganglion cells are now believed to function as the primary photoreceptors for this system, since the sensitivity, spectral tuning, and kinetics of the response to the light fit with those of the light entrainment [6].

### 2.2. The Core Clock Machinery

Since those discoveries great progress was made in this research field, and thanks to the efforts of a few pioneers who worked on it from the middle of 1960s’, we now know quite a lot of how our internal clocks are organised and how deeply rhythmic we are.

The most important breakthrough was the discovery of the anatomical localisation of the clock, and the assessment of which cells and which physiological mechanisms were involved. In 1967 it was demonstrated that after damaging the hypothalamus in rats, the animals lost most of their behavioural periodicity [7]. Just five years later, the exact site of the clock was localised to a small group of cells in the anterior hypothalamus, subsequently termed the suprachiasmatic nucleus (SCN) [8], and the neural connection linking the eye to the SCN was also clarified, and named the “retinohypothalamic tract” (RHT) [9]. The regulation of the internal periodic behavioural patterns driven by the SCN was recognised as an intrinsic physiological property of the SCN itself, namely its ability to generate a rhythmic electrical activity with a period of about 24 h. Such an electrical oscillation in turn induced a similar pattern in the neighbour neurons, even if with a phase shift in nocturnal animals (e.g., rats) during the night [10,11,12].

The molecular machine responsible for the functioning of the circadian clock was unravelled after a series of experiments, using mutants of the fruit fly *Drosophila*. The first gene identified was named *period (Per)*, because the mutant flies had an altered eclosion time [13]; the protein codified by this gene (PER), showed rhythmic cycles of synthesis well-coordinated with the circadian behaviour of the fly [14]. The model explaining those observations was proposed several years later, and it was based on a complex negative feedback loop, which involves, in addition to PER, three other proteins: (i) the product of the *timeless (Tim)* gene, TIM, also identified in *Drosophila* [15,16]; (ii) CLOCK, whose gene *Clock*, was originally identified in mice [17]; and (iii) CYCLE (CYC). The regulation is achieved through the formation of a complex CLOCK•CYCLE, which binds to the E-box region on the *Per* and *Tim* promoters, activating their transcription. When then levels of PER and TIM increase, the complex PER•TIM binds to CLOCK•CYCLE, preventing its role as a transcriptional activator until the transcription is completely abolished, and a cycle of synthesis can start again. This mechanism is further fine-tuned by the protein kinase Doubletime (DBT), which phosphorylates PER, targeting it for proteasomal degradation. DBT can phosphorylate PER only in its free form, and not when it is complexed with TIM; for this reason, only when the levels of TIM increase, the complex PER?TIM may form, and inside the nucleus its stability is about 10 h. The entrainment by light is achieved by two different systems that perceive the luminous input, one through the photopigment cryptochrome (CRY), the other through opsin-based photopigments. Once the light has been sensed, the stimulus results in TIM degradation, which in turn will reset the whole mechanism [18].

Although the general outline of the clock is similar to that operating in *Drosophila*, in mice some key differences exist. Three Per homologue genes were identified until now, named *mPer1-3*, whose transcription is rapidly stimulated by light, with a period of about 24 h [19,20,21]. Up to now there are no clues of the existence of a gene performing the same function as *Tim* in *Drosophila*, even if the central role of one of these possible analogues in entraining the clock in cooperation with *Per1/2* was suggested [22]. Interestingly, it was shown that *Tim* plays a key role in the regulation of developmental processes. For example, in mouse and rat embryos *mTim* was highly expressed in the developing lung, liver, and kidney, as well as neuroepithelium, strongly supporting its role in epithelial organogenesis [23]. Moreover, it was recently assessed that *mTim* plays a role in the regulation of apoptotic processes that coordinate the differentiation of mouse embryonic stem cells, thus corroborating the hypothesis of a deep relationship between the circadian clock and the early developmental stages in mammals [24]. The analogue of *cycle* was identified, and named *Bmal1* in mammals [25], whereas, as described previously, *clock* was originally identified in mice [17]. In mammals *Cry* is not involved in the reception of light, but it nevertheless represents an essential part of the feedback mechanism, with both of its isoforms, *Cry1* and *Cry2* [26,27,28]. The mechanism is further refined by additional components, such as *Rev-Erb* α/β and *ROR* α/β, which bind to the RORE enhancer element in the *Bmal1* gene, and repress and stimulate its transcription respectively. Though previously believed not fundamental for the generation of circadian rhythmicity [29,30,31,32], they were recently recognised to play a more important structural role in the overall functioning of the clock machinery [33]. Finally, the role of DBT in *Drosophila* is achieved in mammals by casein kinase 1ε (CK1ε) and casein kinase 1 δ (CK1 δ), which together (CK1δ/ε) phosphorylate PER proteins, addressing them to proteasomal degradation [11,34,35,36]. Thus, the overall process in mammals starts after the light is sensed by the specific photoreceptor melanopsin, which is localised in the retinal ganglion cells forming the RHT tract and projecting to the SCN [6,37,38,39]. This stimulus triggers a chain of responses that in turn reset the clock by the increase of transcription of *mPer1-3*. The *de novo* synthesised PER proteins, after binding to CRY, enter into the nucleus, and this PER•CRY complex is able to inhibit the transcription of the clock controlled genes (ccg) and of their own genes, usually activated by the binding of CLOCK•BMAL1 to the E-box enhancer elements [40,41].

**Figure 1:**
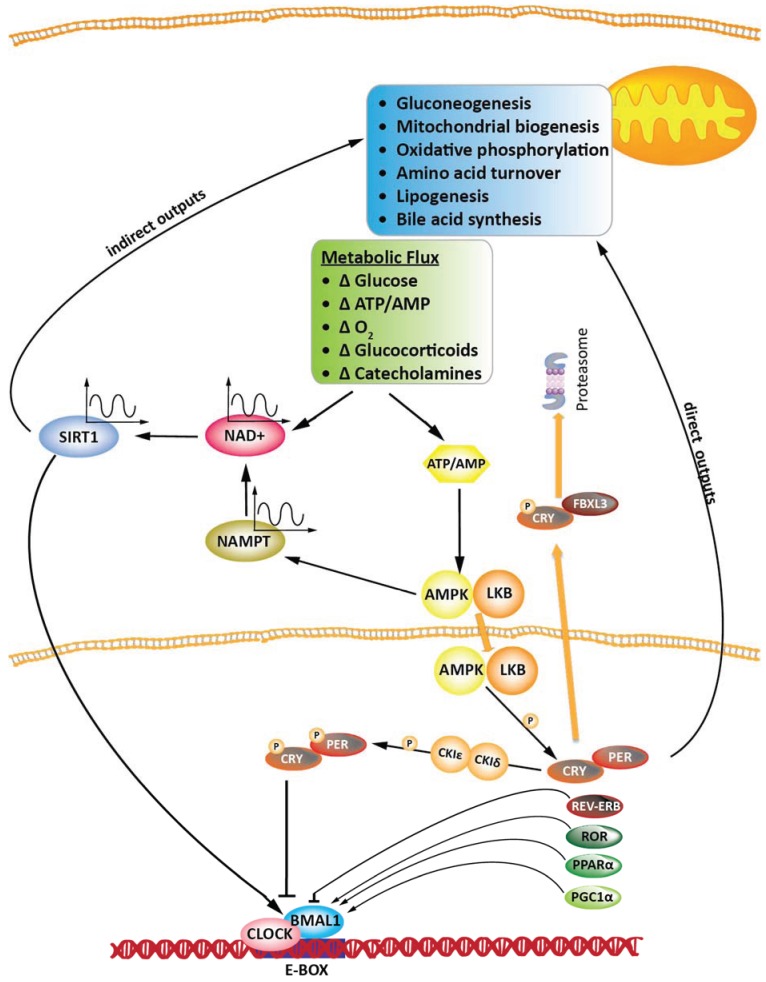
Schematic drawing representing the molecular machine that regulates the circadian clock and its interplay with metabolism. Waves symbolises circadian regulation. NAMPT: nicotinamide phosphoribosyltransferase. See text for details.

As mentioned before, an additional checkpoint is represented by CK1δ/ε-driven phosphorylation of PER•CRY, which targets the complex for degradation by the proteasome. It was determined that CLOCK has a histone acetyltransferase (HAT) activity, and its heterodimeric partner BMAL1 enhanced HAT function. This HAT activity of CLOCK was essential to restore circadian rhythmicity and the activation of circadian genes in *Clock* mutant cells [40]. CLOCK was also able to acetylate BMAL1, which underwent rhythmic acetylation in mouse liver, with a timing that parallels the down-regulation of circadian transcription of clock-controlled genes. BMAL1 acetylation facilitated recruitment of CRY1 to CLOCK-BMAL1, thereby promoting transcriptional repression. Thus, this enzymatic interplay between the two clock components is fundamental for the functioning of the circadian clock machinery [41]. Figure 1 gives a schematic overview of the molecular organisation of the circadian clock and of its main outputs on the regulation of metabolic pathways. The effect of variations of metabolic flux on the circadian machinery is also represented. The role of SIRT1, NAD^+^ and AMPK will be extensively discussed in Section 4 of this review.

The circadian activity generated in the SCN, needs to be transmitted to the rest of the brain and henceforth to the rest of the body. The SCN is able to transmit signals in two principal ways: (i) neuronal networking, by directly interacting with several other brain structures (ii) chemically, by producing diverse signalling molecules. As far as the physical interactions are concerned, the SCN connects directly with the subparaventricular zone (sPVZ) [42], the preoptic area (POA), the bed nucleus of the stria terminalis (BNST), the lateral septum (LS), the dorsomedial hypothalamus (DMH), the arcuate nucleus (ARC), and the paraventricular nucleus (PVN) [43]. The signal from the SCN to the above-mentioned structures is communicated through classical neurotransmission, mediated by GABA and glutamate [44]. The SCN is also able to produce itself a repertoire of signal molecules, which in turn act on neighbouring structures. Among the best characterised until now there are arginine vasopressin (AVP) [45], vasoactive intestinal peptide (VIP) [46], cardiolipin-like cytokine [47], prokineticin 2 (PK2) [48], and transforming growth factor α (TGFα) [49,50].

#### 2.2.1. SCN as a Regulator of Hormonal Homeostasis

One of the first and best-characterised interactions of SCN is with the pineal gland through the sympathetic neurons of the superior cervical ganglion [51]; in turn, the rhythmic activity of the SCN determines the release of melatonin, which directly correlates with the day length. This communication is very important in the regulation of secretion of several key hormones, such as gonadotropin- releasing hormone (GnRH), luteinising hormone (LH), and the follicle-stimulating hormone (FSH) [52]. Melatonin is involved in the regulation of circadian and circannual rhythms in all tissues and hence, from several points of views it can be regarded as a real “zeitgeber”. Synthesis of melatonin also occurs in peripheral tissues, such as the gastrointestinal tract, the retina, skin, lymphocytes and bone marrow, from which it may in turn influence other physiological functions through paracrine signalling. Indeed, melatonin was shown to affect diverse physiological functions, such as regulation of blood pressure [53], modulation of the immune system and free radical scavenging [54].

The circadian activity of the SCN directly affects the rhythmical secretion of several other key hormones. Arginine vasopressin (AVP), or simply vasopressin, produced in the pituitary gland, is able to affect water balance, by decreasing its elimination from kidneys to prevent dehydration. The SCN directly regulates its circadian liberation into the cerebrospinal fluid [55], and as reported before, AVP is produced by the SCN itself, where it is essential for the synchronisation of the neuronal activity [56]. The other key molecule that shows a circadian pattern of release is the neurotransmitter acetylcholine (ACh) [57], which in addition to the well-known effects on muscle contraction and gluconeogenesis, is also able to play a role in the regulation of the firing activity of the SCN, acting through a muscarinic ACh receptor [58]. The periodic release of glucocorticoids from the adrenal cortex is also orchestrated by the SCN, which determines the maximal output in the early morning for diurnal animals and in the early evening for the nocturnal ones [59,60]. Adrenocorticotropic hormone (ACTH) displays a similar pattern of release from the corticotrope cells of the pituitary, and in turn stimulates the release of corticosterone from the adrenal cortex [59]. Remarkably, this process is repressed by light, and it seems to be directly dependent on SCN, through its connections to the paraventricular nucleus [59]. This accurate control is very important considering the central roles played by this hormone, both as a precursor of aldosterone and for its regulation of liver metabolic functions, hence behaving as a glucocorticoid [61]. Notably, the glucocorticoid receptor agonist dexamethasone is able to synchronise circadian gene expression in rat fibroblasts* in vitro* and to shift the phase of expression of circadian genes in liver, kidney, and heart; moreover, due to the absence of glucocorticoid receptors in the SCN, dexamethasone does not change its circadian behaviour [62].

### 2.3. Peripheral Clocks

In addition to being present centrally in the SCN, the machinery responsible for the generation of the circadian activity is expressed in almost all the peripheral tissues, as recently revealed in rodents [63,64]. This peripheral periodicity was dependent on the activity of the central oscillator, for it attenuated until stopping without the contribution of the SCN after 2–7 cycles in liver, lung and skeletal muscle* in vitro* [63]. However, a successive study reported that peripheral tissues were capable of self-sustained circadian oscillations for >20 cycles in isolation. In addition, peripheral organs expressed tissue-specific differences in circadian period and phase, and lesions of the SCN did not block circadian rhythmicity, but instead just desynchronized the peripheral tissues of individual animals and from different animals. So, peripheral organs express at least a partially self-sustained circadian oscillator [64]. Such circadian behaviour was shown also* in vitro* in several cell types [65,66,67,68], and in tissue explants from almost all organs [64,69]. Interestingly, the brain itself possesses its own circadian oscillation in the expression of several genes that seems to be independent of the activity of the SCN, at least according to what was shown in the olfactory bulb [70,71,72].

About 2% to 10% of the whole genome showed a circadian pattern of expression in several tissues as measured in mice [73,74,75,76]. Some of these genes were expressed in a tissue-specific manner, but the vast majority was common for almost all tissues; the genes implicated in the periodicity—as for example *Per2*, *Bmal1*, *Rev-erbα* and *Cry*—displayed the highest degree of conservation [77].

#### 2.3.1. Entrainment of Peripheral Clocks

The peripheral clocks are synchronised by three main sources of entrainment: (i) direct entrainment through neural and hormonal signals (controlled by the SCN); (ii) food entrainment; and (iii) body temperature entrainment.

Neural control is achieved through the autonomic nervous system, whose outputs are in turn indirectly controlled by the SCN. For example, in SCN-deficient rats, the light did not drive the sympathetic-induced release of corticosterone by the adrenal gland [78]; analogously, in rats without the SCN, the hyperglycaemic effect of GABA antagonists was lacking [79]. In rats whose autonomic liver innervation was surgically impaired, the light did not induce the upregulation of *Per1*/*2*, *Pepck* and *Glut2* [80]. This finding further supports the key-role of the autonomic nervous system.

The activity of the SCN is also able to regulate the secretion of several essential hormonal mediators, such as AVP, Ach and glucocorticoids, as discussed previously in Section 2.2.1.

Food exerts an important entrainment role in several peripheral organs, such as heart, kidney, pancreas and liver, as we will examine in a more detailed way in the following sections. In the mouse liver most of the genes that cycle in a circadian way were directly involved in the regulation of metabolic pathways [81,82], similarly to what was observed in the heart [74]. In the liver and in lung fasting-feeding cycles operate strongly in the synchronisation of the peripheral clock. In rats, which typically have a nocturnal feeding behaviour, the inversion of feeding cycles—thus artificially inducing a diurnal eating—rapidly and extensively changed the hepatic expression of metabolic genes; rhythmicity in the lung was also slightly affected [83]. Importantly, the SCN seems to play a multifaceted role in the time-setting of the different peripheral organs, as was clarified in mice with damaged SCN. These animals still showed a regular periodicity in liver and kidney, but not in skeletal muscle, heart and spleen [84]. These findings further underline the complexity of the system, and the interplay between different regulatory mechanisms. Moreover, in *Cry1/2*-deficient mice a temporally restricted feeding protocol was able to restore the circadian transcriptional periodicity of the vast majority of the hepatic genes. Conversely, without a fixed schedule in feeding, the animals kept the transcriptional periodicity of just the minor part of the usual circadian-expressed genes [8]. The regulation of circadian rhythms in peripheral tissues by feeding/fasting is achieved also through the release of hormones such as peptide YY, oxyntomodulin, cholecystokinin, leptin, and ghrelin, which directly signal to the neurons of the paraventricular nucleus (reviewed in [85]). The extent of the association of the SCN in this regulation and the mechanisms involved are still under investigation; however it was demonstrated that when the SCN was damaged the circadian patterns of feeding and drinking were abolished [8,86].

The third important factor for the regulation of the circadian clock in the peripheral tissues is temperature, even if the mechanisms involved are up to now not fully understood. The SCN itself is able to compensate temperature variations of the external [87,88,89] and internal environment [87]; this process seems to be mediated by CRY and PER [90,91]. On the other hand, the SCN activity regulates daily changes in body temperature [92], and these changes in turn were shown to keep the peripheral clocks synchronised [89]. In *Drosophila*, in addition to light, temperature mediated the interaction between CRY and the complex PER•TIM. Heat-induced phase shifts were severely reduced in *cry* loss-of-function mutants, and *per* mutants showed a significant enhanced temperature sensitivity of biochemical interactions and behavioural phase shifting. Light and temperature acted together to affect the rhythms in wild-type flies through similar mechanisms, since the interaction between CRY and the complex PER•TIM was central for circadian responses to both light and temperature variations [90]. Thus, working synergistically to orchestrate the circadian phase, light and temperature could fine-tune the regulation of the clock, hence contributing to the seasonal adaptations of clock function [90].

#### 2.3.2. Liver and Pancreas Clocks

The liver is a key organ in the control of lipid and glucose homeostasis, bile acid synthesis, and cholesterol, amino acids and xenobiotic metabolism [74,75,93]. As already mentioned in the previous sections, the levels of expression of hepatic genes follow a circadian periodicity, for both clock-related and hepatic-specific genes. The circadian genes seem to be fundamental for the preservation of the liver functions, as demonstrated in *Bmal1*-deficient mice, which lost their rhythmical behaviour either in brain and in the liver, and in *Clock*-deficient mice, whose hepatic circadian clock was abolished but still retained central periodicity [17,75]. Nonetheless, liver periodicity seems to possess a relative autonomy from the central regulator, as assessed in mice; in fact, here the glucocorticoid receptor—a powerful transcriptional activator—was able to rescue about 60% of the circadian gene expression that was shut off by damaging the SCN [61]. Moreover, the liver oscillator is strongly dependent upon food for tuning, even if the central pacemaker is correctly working, as more than the 80% of the hepatic transcriptome is “meal-dependent” [8].

The pivotal role of the circadian oscillator in the regulation of glucose metabolism was revealed in *Bmal1*-deficient mice. Here the liver-specific inactivation of *Bmal1* resulted in severe hypoglycaemia during their inactivity period, but not if *Bmal1* was inactivated in all the other cell types excluding the liver; comparable results were obtained after inactivation of *Per1/2* [94]. From what has emerged from a recent study, *Per2* plays a role in the regulation of glucose homeostasis also in humans [95].

In mice, the regulation of bile acid and cholesterol biosynthesis is under circadian control through *Rev-Erb*α, which regulates the expression of SREBP, and consequently that of cholesterol metabolism genes. It was also supposed that the cyclic expression of cholesterol-7α-hydroxylase (*Cyp7a1*) might be driven by a REV-ERBα mechanism, by means of an oxysterol-mediated LXR activation [96].

The role of the pancreas in maintaining the glucose balance is well known, and recently the presence of an independent circadian oscillator, essential in driving the proper functioning of pancreatic functions, was clarified [97,98]. The fundamental role is played by *Bmal1* and *Clock*, whose selective knockdown in mice led to modifications of the proliferative rate and size of islet cells, together with hypoinsulinaemia, reduced glucose tolerance and diabetes. Remarkably, in the same mouse system, the re-establishment of the pancreatic islet function results in an improvement of the insulin resistance, but not of obesity [99].

## 3. The Circadian Clock in Lipid Metabolism and Transport

### 3.1. Circadian Regulation of Triglycerides Levels

Evidences showing that the levels of triglycerides in the plasma oscillate following a circadian pattern were provided long ago in both humans [100] and rodents [101], in these latter with a maximum during their activity phase. In humans two separate spikes in plasma triglycerides levels were identified: the first, smaller, around eight hours after arousal, and the second about 20 hours after awakening. Desynchronisation of the sleep schedule did not affect the appearance of the first peak, but resulted in the loss of the second one. It was thus hypothesised that the first one was mostly dependent upon an endogenous circadian regulation, and the second probably more finely-tuned by the sleep cycles [102]. By means of a metabolomic approach [103], it was demonstrated that in humans around 15% of all the small molecular weight metabolites taken into account showed circadian variations and among them more than 75% were lipids, mostly fatty acids. From this work it emerged that the endogenous circadian clock is the principal regulator of these fluctuations in the plasma lipid profile, as changes in the sleeping or feeding schedule could not affect this pattern [103]. A successive study shed light on which was the chemical nature of the cycling fatty acids in the plasma, and the major part was constituted by diacylglycerols and triacylglycerols [104].

Several lines of evidence have shown that the molecular clock is directly involved in the control of lipogenesis. Mice with a mutated *Clock* gene were prone to develop hypertriglyceridaemia [105], and this was later ascertained to be primarily due to effects at the liver and enterocyte level [106]. A key role in the regulation of lipid metabolism was determined for *Nocturnin*, a gene expressed in a circadian manner, whose product is an enzyme with a deadenylase activity. *Nocturnin*-deficient mice on a high-fat diet did not increase their body weight and visceral fat, and did not develop fatty liver, without increasing their activity rate or decreasing food intake. The authors suggested that *Nocturnin* could affect lipid metabolism or uptake, together with glucose and insulin sensitivity [107]. *Clock*-deficient mice displayed also a reduced hepatic triglyceride accumulation under high-fat diet conditions, due to the suppressed expression of the two key genes *Acsl4* and *Fabp1* [108].

### 3.2. Circadian Regulation of Lipid Transport

#### 3.2.1. Periodicity in the Intestine

Similarly to the organs described previously, the intestine shows a circadian periodicity in several of its functions, such as DNA synthesis, epithelial cell renewal, electrolyte and food absorption, food-anticipatory activity, motility [109,110,111,112,113]. In rodents, the maximal expression of clock-related genes was observed during the day in both the colon [110,114] and the jejunum [112]; these spikes were coordinated with those present in the liver, but phase-shifted with respect of those in the SCN [83]. In humans, deleterious effects on gastrointestinal functions were reported due to periodic or chronic sleep deprivation or disturbances, as well as to periodic misalignment of the circadian rhythm, as it occurs in frequent long trans-meridian voyages [115]. The spectrum of the pathologies described is wide, including peptic ulcer, gastro-oesophageal reflux and irritable bowel syndrome, up to risks of polyps and colorectal cancer [116].

#### 3.2.2. Lipid Transport: A Short Overview

The digestion of the main dietary lipids,* i.e.* cholesterol esters, phospholipids and triglycerides, takes place in the intestinal lumen, where they are emulsified by bile salts and incorporated into bile salt micelles [117,118]. Triglyceride digestion begins in the mouth by means of salivary lipases, then follows in the stomach by gastric lipases, and is finished in the small intestine by pancreatic lipases, whose enzymatic activity produces free fatty acids (FFAs) and monoacylglycerols. Pancreatic phospholipases are the main phospholipid-hydrolysing enzymes, and among them phospholipase A2 is the principal one, which produces FFAs and lysophospholipids; likewise, cholesterol esterases produce FFA and free cholesterol. The absorption of FFAs and monoacylglycerols into the enterocyte takes place by diffusion or by means of protein transporters such as clusters of differentiation 36 (CD36). Several cholesterol transport proteins have been identified up to now, such as Niemann-Pick C1-like 1 (NPC1L1), scavenger receptor B1 (SRB1), and CD36. All these aspects have been extensively reviewed [119].

The processing of FFAs follows in several organelles, but principally inside the endoplasmic reticulum (ER), where they are transported by means of specific FA-binding proteins. The ER has all the enzymatic activities needed to synthesise phospholipids, cholesterol esters and triglycerides. Phospholipids and triglycerides are synthesised from monoacylglycerols through different pathways. Inside the enterocyte, monoacylglycerol acyltransferase synthesizes diacylglycerol (DAG) from FFA, and monoacylglycerol and diacylglycerol acyltransferases (DGATs) release triglycerides. On the other hand, ethanolamine and choline transferases are mainly responsible for phospholipid biosynthesis from ethanolamine and choline, and DAG. The esterification of free cholesterol is performed by an acyl-CoA:cholesterol acyltransferase [119].

Then lipids are packed inside the chylomicrons (CMs), which usually contain phospholipids, mainly on the surface, and triglycerides and cholesteryl esters in the central part. On the surface there are also several scaffolding proteins, named apolipoprotein (Apo); the main Apo component in CMs is ApoB48, together with ApoC, ApoAI and ApoAIV [119,120]. The translation of ApoB48, which takes place on the ER membrane, is stringently dependent upon the presence of microsomal triglyceride transfer protein (MTP) and of a sufficient amount of lipids. MTP transfers TG to ApoB, helping the formation of a folding structure more favourable to store large amounts of lipids [119,121]. This pathway leads to the production of an “HDL-like” chylomicron, which is first provided with phospholipids and neutral lipids externally, and henceforth is loaded with newly synthesised triglycerides and cholesteryl esters (CE) [119,122,123].

Along with triglycerides, CMs carry also relevant amounts of cholesterol; however, free cholesterol can be also transported back to the intestinal lumen by means of the ATP-binding cassette G5 and 8 (ABCG5 and ABCG8), and can be secreted from the basolateral side through the HDL pathway, which involves ApoAI and ABCA1 [124,125]. This transporter plays a very important role for cholesterol transport through the HDL pathway, even if it is not involved in the CMs pathway [126].

#### 3.2.3. Circadian Influence on Lipid Absorption and Transport

In mice lipid absorption at the enterocyte level was higher during the night and lower during the day [112,127,128,129]. Two studies reported circadian oscillations in the uptake and secretion of fatty acids (FAs) and cholesterol, contextually showing the same rhythmical pattern in the mRNA levels of several of the proteins involved in such processes, such as stearoyl-CoA desaturase-1 (SCD-1), fatty acid synthase (FAS), DGAT2, MTP, ApoB and ApoAIV [127]. The expression of these genes was food-entrainable, and the authors also suggested that the genes involved in lipid absorption could be classified as light responsive, food responsive, light and food responsive, and nonresponsive [112].

*Clock* plays a pivotal role in controlling several steps of these processes. Circadian rhythm genes were normally expressed in the enterocytes with the usual circadian pattern that is also food-entrained; this regulation was completely lost in *clock*-mutant mice whose food clock regulation was impaired [127,128]. This mouse genotype showed hypertriglyceridaemia that was mainly associated to their increased lipid absorption during the day; in contrast, wild type animals had the peak of absorption in the middle of the night [112,127,128]. *Clock*-mutant mice were utterly lacking the circadian regulation of *Mtp* expression, which resulted in a nadir of transcription in the middle of the day and in a zenith in the middle of the night. Such a loss of regulation was mainly due to the missed control of the normal diurnal *Clock*-induced repression of the *Mtp* gene, which is usually achieved by means of the synthesis of the transcriptional repressor SHP (small heterodimer partner). Due to this missed *Mtp* repression, *clock*-mutant mice showed a high MTP expression, an increased production of triglyceride-rich lipoproteins and a sustained hypertriglyceridaemia [128].

*Clock* is very important in the regulation of cholesterol metabolism as well, for *Clock*-/*ApoE*- double mutant mice were hypercholesterolemic, due to the accumulation of ApoB48-containing cholesteryl ester-rich lipoproteins, and the increased intestinal cholesterol absorption contributed further to hypercholesterolemia. In *Clock-/-ApoE-/-* mice intestinal expression of Niemann-Pick C1-Like 1 (NPCL1), Acyl-CoA:Cholesterol acyltransferase 1 (ACAT1) and MTP was elevated and the enterocytes assembled and secreted more CMs. Moreover, a macrophage dysfunction was identified as another possible cause of increased atherosclerosis in such double-mutant mice, since they absorbed more lipids if compared with the *ApoE* mutants, and cholesterol efflux from macrophages was impaired, due to the reduced expression of ABCA1 [129].

In humans under a standard diet regimen, apoB48 showed a diurnal variation with three distinct spikes, the first around 10:00 a.m., the second at about 14:00, the last between 18:30 and 19:00. ApoB100 did not show any variation, not even after a fat load, suggesting that its synthesis is not subjected to entrainment by food [130].

#### 3.2.4. Circadian Control of Lipid Biosynthesis

Circadian genes do not only control lipid absorption, but are also involved in the regulation of lipid biosynthesis. It was long ago observed in rodents that cholesterol synthesis showed a circadian pattern in the liver and in the intestine, being higher during the night and lower during the day [131,132,133,134,135,136,137,138]. This regulation was mainly achieved through the circadian expression of β*-hydroxy-3-methylglutaryl-CoA reductase* (HMG-CoA reductase) and of *Cyp7A1* [131,132,133,134,135,139]. Together with light entrainment, cholesterol biosynthesis and *HMG-CoA reductase* expression appeared to be strongly entrained also by food ingestion, as shown in several rodent models in both the intestine and the liver [133,134,135,140], and also in a human study [141]. In rodents bile acid (BA) profile followed a circadian periodicity in the serum, liver, gallbladder and intestine; the zenith and nadir of these fluctuations were the opposite of those observed for cholesterol biosynthesis in the serum and intestine, but not in the liver and gallbladder [142,143]. In one human study, BA synthesis respected a circadian schedule, with two main peaks during the day, one around 1 p.m., the other round 9 p.m. On the other hand, the circadian rhythm of cholesterol biosynthesis showed a nocturnal peak, between 12 and 4 a.m., exactly the opposite of what was described in rodents. The patients involved in the study had primary endogenous hypertriglyceridaemia, and were kept on a carbohydrate-rich diet (meals at 9:00, 13:00 and 17:00 h). Serum TG showed a wavelike pattern with the maximum at around 17:00 h, differently to normal subject that peaked at around 14:00 h. In the fasting state, the activity of lipoprotein lipase (LPL) was not different from that observed in healthy subjects, but did not show the normal rise in the fed state (16:30 h). This was determined by the missed increase in the activity of the adipose tissue (AT)-LPL during the day, which was always lower than in normal subjects. LPL activity in skeletal muscle was always low, without any diurnal change. Low-density lipoprotein cholesterol and high-density lipoprotein (HDL) cholesterol concentrations did not vary during the day, but HDL phospholipids showed a significant increase during the day [144]. In mice, the diurnal variations of BA concentrations and composition might play important roles in coordinating daily nutrient absorption and energy homeostasis. This regulation was achieved by influencing the circadian rhythms of expression of BA metabolising genes in the liver and ileum and of enterohepatic genes encoding key proteins involved in BA biosynthesis and transport [145]

The levels of several key proteins implicated in the regulation of triglyceride metabolism—such as lipolytic enzymes, ApoAIV and PPARα—oscillated with a circadian periodicity, both in rodents and humans [137]. In mice fatty acid synthase (FAS), acetyl-CoA carboxylase (ACC), SREBP1c, and fatty-acid-binding protein 4 (FABP4) fluctuated in a circadian way in both adipose tissue and liver [108,146].

Several authors have recently supported a pivotal role of *Clock* in the regulation of both cholesterol and triglyceride metabolism. In wild-type mice, the hepatic circadian regulation of *HMG-CoA reductase*, low-density lipoprotein receptor (*LDLr*), and *Cyp7A1* was dampened and finally abolished after four weeks under a cholic acid diet. *Clock*-mutant mice, on the other hand, in addition to an unscheduled expression of clock-genes, such as *Bmal1* and *Per2*, showed also an arrhythmic expression of *HMG-CoA* *reductase*, *LDLr* and *Cyp7a1*. Moreover, when fed with a high cholesterol+cholic acid diet, these mice displayed a remarkable cholesterol accumulation in the liver and a reduced expression of *Cyp7a1* with respect to wild-type mice [147]. *Clock*-mutant C5B1/6J mice were obese and showed all the typical characteristics of metabolic syndrome, such as hyperglycaemia, hypertriglyceridaemia, hypercholesterolemia and hypoinsulinaemia [105]. Differently, in *Clock*-mutant ICR mice, serum levels of triglycerides and free fatty acid were significantly lower than in wild-type control, whereas total glucose and cholesterol levels were unchanged. Likewise, in homozygous *Clock*-mutant mice the increase in body weight induced by a high-fat diet was reduced, and dietary fat absorption was severely impaired. Interestingly, in the pancreas of *Clock*-mutant mice, circadian expression of cholecystokinin-A (CCK-A) receptor and lipase mRNAs was greatly reduced [148]. The role of *Clock* in the regulation of the lipid metabolism seems thus to be quite complex, with several points that need to be clarified in the future.

*Clock* was not only able to affect deeply the regulation of triglyceride and cholesterol metabolism, but high fat feeding and metabolic alterations influenced its expression. C5B1/6J mice on a high-fat diet showed a reduced circadian pattern of expression of clock and clock-controlled genes in the adipose tissue and in the liver [146]. On the contrary, ICR mice fed a high-fat diet displayed symptoms of metabolic syndrome with hyperglycaemia, hyperlipidaemia and obesity. Nonetheless, the high-fat feeding was almost ineffective in changing the rhythmic expression of the clock genes examined (*Clock*, *Bmal1*, *Per1*, *Per2*, *Cry1*, *Cry2*) in both the liver and visceral adipose tissue. Mice on high-fat diet, however, completely lost the circadian control of the expression of *Cyp7A1*. High-fat feeding and mild metabolic syndrome seemed thus to not directly affect the molecular clock system, but instead to interact with the circadian expression of metabolism enzymes [149]. Again, more studies will be required to clarify how fat accumulation and metabolic diseases are able to interfere with the circadian clock machinery.

Some light was recently shed on the deep relationship between the circadian clock machinery and lipid metabolic pathways. BMAL1 turned out to be a key factor in the regulation of adipogenesis and lipid metabolism in mature adipocytes, and it is also important for adipocyte differentiation, since *Bmal1* knockout mice embryonic fibroblasts failed to differentiate into adipocytes. *Bmal1* expression in 3T3-L1 adipocytes triggered the expression of several lipogenic factors, as the promoter activity of these genes was stimulated in a BMAL1-dependent manner. The expression of these factors was clearly circadian in mice adipose tissue, and the overexpression of BMAL1 increased lipid biosynthesis [150]. The circadian clock was also directly involved in the regulation of the expression of the transcriptional regulator Spot14, which controls the genes implicated in fatty acid synthesis, being activated in response to lipogenic stimuli. Hepatic levels of *Spot14* mRNA peaked at the early dark period, the usual rodent feeding-time. Under fasting, the concentration of *Spot14* mRNA was commonly diminished, but the periodicity was still retained, implying both a nutritional- and circadian clock-mediated entrainment. The circadian expression of *Spot14* was kept also under constant darkness and the rhythmicity was absent in *Clock* mutant mice. Hence, a synergy between the light entrainable and the food entrainable oscillators, and food-derived nutrients, controlled the circadian expression of *Spot14* in the liver [151].

The transcriptional coactivator peroxisome proliferator-activated receptor-gamma coactivator 1β (PGC-1β), plays a central part in the control of the expression of several nuclear-encoded genes regulating mitochondrial and metabolic functions in multiple tissues—including brain, brown adipose tissue, skeletal muscle, heart and liver—but is also involved in the proper functioning of the circadian machinery. In fact, in contrast to *Pgc-1*α KO mice that were hyperactive, *Pgc-1*β KO mice displayed a significantly decreased activity during the dark cycle [152]. The nuclear receptor REV-ERBα, which is directly involved in the control of circadian rhythms, similarly plays a regulatory role in lipid metabolism and adipogenesis, and in the control of BA metabolism through the regulation of the neutral bile acid synthesis pathway. *Rev-erb*α-deficient mice showed a lower synthesis and an impaired excretion of BA into the bile and faeces, and a decreased hepatic expression of *Cyp7A1* [153].

PER2 was likewise involved in the regulation of lipid metabolism and it directly repressed *Pparγ* expression, a nuclear receptor critical in adipogenesis, insulin sensitivity, and the inflammatory response. *Per2*-deficient mice had altered lipid metabolism with a severe reduction of total triglycerides and non-esterified fatty acids; PER2 blocked PPARγ-mediated transcriptional activation by impeding recruitment to target promoters. *Per2*-deficient cultured fibroblasts showed enhanced adipocyte differentiation, and lipidomic-profiling studies demonstrated that it was required for normal lipid metabolism in white adipocyte tissue [154]. The existence of more structured machinery behind the circadian regulation of lipid metabolism was very recently suggested. In this study a broad lipidomic analysis of hepatic tissue from *Per1/2* null mice revealed that around the same amount of lipids (about 17%) oscillated both in the mutant and in the wild-type mice, but following different phases and with different ratios between the oscillating species. Interestingly, several key enzymes of triglyceride metabolism were expressed in a circadian manner, even in *Per1/2* mutants, and the feeding time played a central role in orchestrating the daily triglyceride metabolism both in the mutant and in the wild-type mice. Thus, it is plausible to suppose that more complex systems work in the regulation of daily triglyceride profile [155].

**Figure 2:**
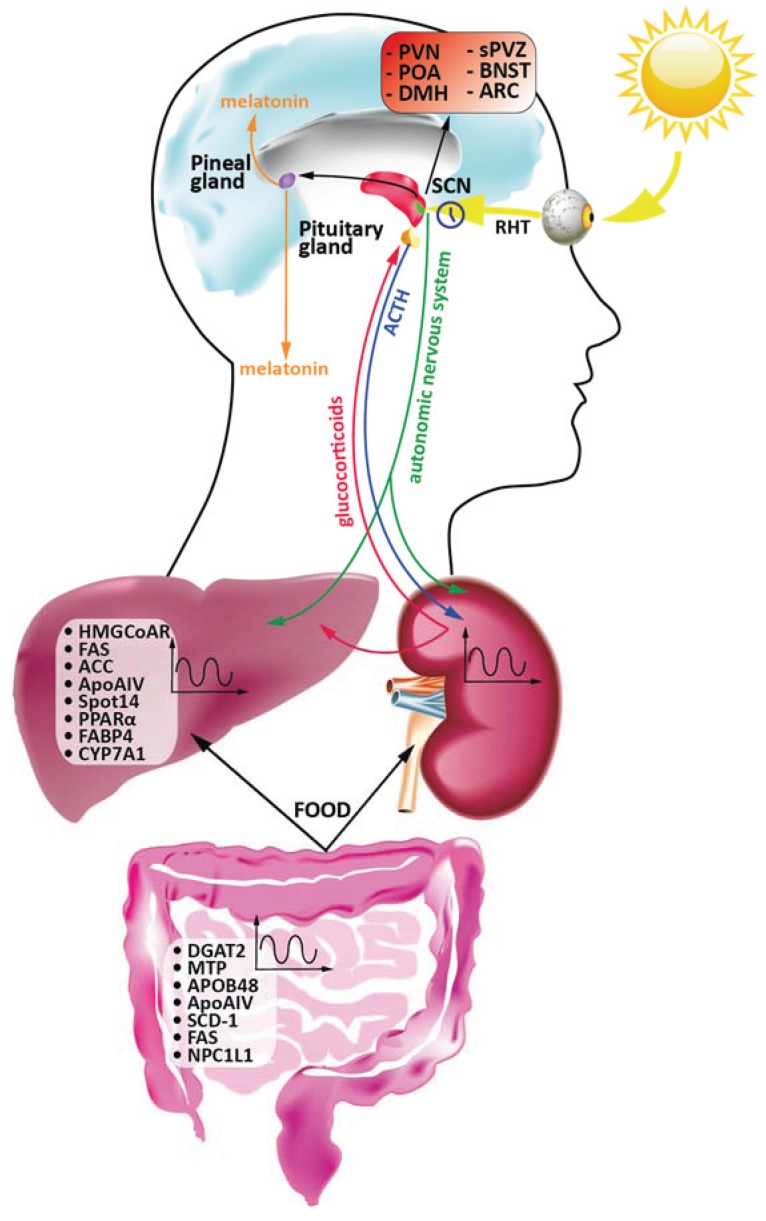
Schematic overview of the interplay between the central and peripheral circadian clocks. Waves symbolises circadian regulation. RHT: retinohypothalamic tract; SCN: suprachiasmatic nucleus; sPVZ: subparaventricular zone; POA: preoptic area; BNST: bed nucleus of the stria terminalis; ARC: arcuate nucleus; DMH: dorsomedial hypothalamus; PVN: paraventricular nucleus; ACTH: adrenocorticotropic hormone; HMGCoAR: HMG-CoA Reductase; FAS: Fatty Acid Synthase; ACC: acetyl-CoA carboxylase; ApoAIV: apolipoprotein AIV; FABP4: fatty-acid-binding-protein 4; CYP7A1: cholesterol-7α-hydroxylase; DGAT2: diacylglycerol acyltransferase 2; MTP: microsomal triglyceride transfer protein; SCD-1: stearoyl-CoA desaturase-1; NPC1L1: Niemann-Pick C1-like 1. See text for details.

Figure 2 summarises what has been analysed in detail in text about the interplay between the central and peripheral circadian clocks, underlining the genes under circadian control in the liver and intestine.

## 4. Circadian Clock in the Regulation of Cellular Metabolism: SIRT1, AMPK, PGC-1α, cAMP

In the last two decades understanding of the mechanisms that govern the internal periodicity of higher organisms was greatly improved, and now we understand more in depth the circadian basis by which metabolism is regulated [156,157,158,159]. New interesting avenues have been recently opened that expand the intermingling between the regulation of metabolism and our molecular clock.

A central role in this sense was addressed to SIRT1, a member of the SIRTs family of histone deacetylases, whose activation has been associated with panoply of beneficial effects as a positive regulator of metabolic homeostasis [160]. SIRT1 is a class III histone deacetylase whose enzymatic activity, differently to that of class I and II, depends upon the presence of NAD^+^ as a cofactor; hence, during fasting, when the concentration of NAD^+^ is increased, the activity of SIRT1 is elevated [161]. SIRT1 was shown to directly modulate the rhythmic expression of several circadian controlled genes. Systematic NAD^+^-dependent deacetylation by SIRT1 of histones, BMAL1, and finally PER2 facilitated the establishment of a repressive chromatin state [162]. Moreover, the HDAC activity of SIRT1 was regulated in a circadian manner, linked with the rhythmic acetylation of BMAL1 and histone H3 at circadian promoters. SIRT1 associates with CLOCK and is recruited to the CLOCK•BMAL1 chromatin complex at circadian promoters, and the genetic ablation of *Sirt1* or pharmacological inhibition of SIRT1 activity resulted in disruptions of the circadian cycle and of the acetylation of H3 and BMAL1: hence SIRT1 might function as a controller of circadian machinery, detecting changes in cellular metabolites [163]. Interestingly, intracellular NAD^+^ levels exhibited circadian oscillations, due to the circadian expression of NAMPT (nicotinamide phosphoribosyltransferase) mediated by CLOCK•BMAL1 [164]. SIRT1 was therefore recruited to the *Nampt* promoter, controlling the circadian synthesis of its own coenzyme [167]. Finally, SIRT1 was necessary for circadian transcription of several key clock genes, such as Bmal1, *Per2*, *Cry1*, and *Ror*γ. SIRT1 bound CLOCK•BMAL1 in a circadian manner and promoted the deacetylation and degradation of PER2. Since its deacetylase activity is dependent upon NAD^+^ levels, SIRT1 probably acts as a molecular link between cellular metabolism and the circadian fundamental machinery (Figure 1) [165].

AMPK is key multi-protein complex shown to be deeply involved in the regulation of metabolism as a general activator of catabolic pathways—glucose uptake, glycolysis, fatty acid oxidation—and inhibitor of anabolic ones—fatty acid and cholesterol biosynthesis. Recently, its direct association with the regulation of the circadian clock was demonstrated, since it is itself subjected to circadian oscillations. One of its regulatory subunits, ampkβ2* (PRKAB2)*, showed a circadian expression, which resulted in a periodic translocation in the nucleus, where it directly phosphorylated CRY1 in association with LKB kinase. Such phosphorylation results in the interaction between the phosphorylated CRY1 and the cofactor FBXL3; the complex is finally degraded into the proteasome (Figure 1) [166]. Among other recently identified mediators of the circadian control of energy metabolism, the transcriptional coactivator PGC-1α plays an important role. PGC-1α is generally involved in the activation of catabolic pathways, and it is activated by several stimuli, interestingly also by SIRT1 and AMPK. PGC-1α was found to be rhythmically expressed in the liver and skeletal muscle in mice and to stimulate the expression of clock genes, such as *Bmal1* and *Rev-erb*α, by co-activating the ROR family of orphan nuclear receptors. *Pgc-1*α-deficient mice had abnormal diurnal rhythms of activity, body temperature and metabolic rate due to the abnormal expression of clock genes and of those involved in energy metabolism (Figure 1) [167].

The cyclic nucleotide adenosine 3',5'-monophosphate (cAMP) plays a central physiological role in the regulation of energy metabolism, being activated by glucagon and inhibited by insulin. It was shown that cAMP signalling plays also a part in the regulation of the oscillatory network, since its signalling was rhythmic and involved in keeping the transcriptional loop in the SCN, setting amplitude, phase, and period. Moreover, its role extended in peripheral mammalian tissues and cell lines as well, supporting the progression of transcriptional rhythms [168]. PARP-1 is an NAD^+^-dependent ADP-ribosyltransferase that controls cell progression and metabolic homeostasis. In the liver the activity of PARP-1 oscillated in a circadian manner and was regulated by feeding. PARP-1 was able to bind CLOCK at the beginning of the light phase and to poly(ADP-ribosyl)ate it. The absence of PARP-1 enhanced the binding of CLOCK•BMAL1 to DNA, leading to a shift of the interaction of CLOCK•BMAL1 with PER and CRY, resulting in the alteration of the CLOCK•BMAL1-dependent gene expression. PARP-1 is thus essential in food entrainment of peripheral circadian clocks and connects feeding with the circadian clock [169].

BMAL1 and CLOCK are also major factors in the regulation of glucose and triglyceride homeostasis, as gluconeogenesis was abolished by deletion of *Bmal1* and depressed in *Clock* mutants, even if the response of glucagon and corticosterone to insulin-induced hypoglycaemia was retained. Additionally, a high-fat diet increased circadian variation in glucose tolerance and insulin sensitivity, and mutations of *Clock* were able to restore the chow-fed phenotype [170]. Circadian clock controlled also hepatic gluconeogenesis, which during fasting was initiated by the cAMP-mediated phosphorylation of cAMP response element-binding protein (CREB). CREB activity was modulated during fasting by *Cry1* and *Cry2*. *Cry1* expression was elevated during the night-day transition, when it reduced the expression of fasting gluconeogenic genes by blocking glucagon-mediated increases in intracellular cAMP concentrations and protein kinase A-mediated phosphorylation of CREB. As hepatic overexpression of *Cry1* lowered blood glucose concentrations and improved insulin sensitivity in insulin-resistant mice, molecules able to enhance cryptochrome activity might theoretically be useful to cope with type 2 diabetes [171].

The deep relationship between the circadian clock machinery and metabolism was further corroborated by the discovery that the highly metabolically-controlled transcription factor REV-ERBα was also of key importance in the synchronisation of the circadian clock, and synthetic REV-ERBα agonists were recently proposed as positive regulators of both metabolism and the circadian clock [172,173]. Other points of contacts between the circadian clock and metabolism, and in particular with the development of metabolic diseases were found, and for example, the activity of the stomach cells that secrete ghrelin was entrained by food through a clock-mediated mechanism [174].

## 5. Conclusions

Circadian rhythms are now recognised as an essential component of the complex physiological machinery that regulates the deep functioning of all organisms, from plants to mammals. The inseparable link between the circadian clock and energy metabolism has been unravelled in the last years, showing how the central clock is able to synchronise the clocks present in the peripheral organs. Up to now, many genes involved in the control of metabolism have been shown to be under circadian regulation, and hence any perturbation of this delicate equilibrium may result in the development of severe pathological conditions. However, several aspects are still under investigation, and possible new discoveries will be helpful not only to increase our scientific knowledge, but also to try to improve public health.

**Figure 3:**
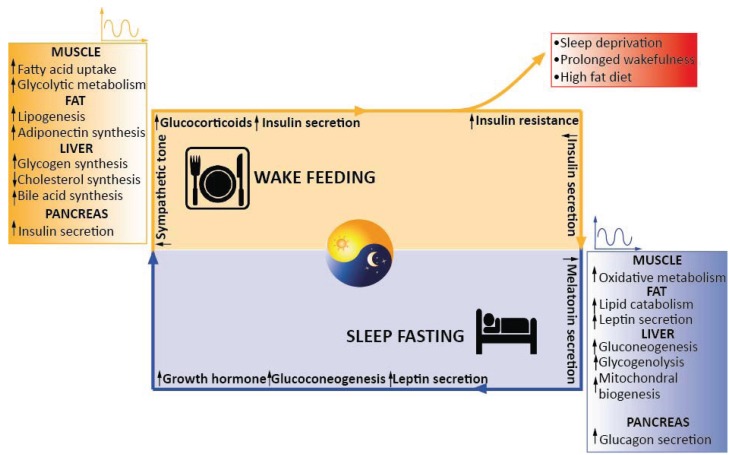
Schematic view of the tight association between circadian rhythms, metabolism and hormonal homeostasis in humans. Waves symbolises circadian regulation. See text for details.

In fact, in the last years, a large part of the general population experienced an increased incidence of sleep reduction and/or a diminished quality of the sleep mainly due to shift work or frequent long transmeridian travels. This disruption of our circadian rhythms was correlated to an augmented incidence of metabolic and cardiovascular diseases (Figure 3) [175,176,177,178,179,180,181]. In this light, new public health policies trying to make larger parts of the population aware of the risks of a bad lifestyle, together with the possible future development of new drugs able to interact directly with the circadian molecular machinery might be beneficial in containing the new rising pandemic in metabolic pathologies.
